# Circular RNA circSHPRH inhibits the malignant behaviors of bladder cancer by regulating the miR-942/BARX2 pathway

**DOI:** 10.18632/aging.203911

**Published:** 2022-02-24

**Authors:** Ling Zuo, Yi Zhu, Jinli Han, Hongwei Liu

**Affiliations:** 1 Department of Traditional Chinese Medicine, The Second Affiliated Hospital of Guangdong Medical University, Zhanjiang 524003, Guangdong Province, China; 2 Department of Urology, Affiliated Hospital of Guangdong Medical University, Zhanjiang 524001, Guangdong Province, China; 3 Department of Urology, Sun Yat-Sen Memorial Hospital, Sun Yat-Sen University, Guangzhou 510120, Guangdong Province, China

**Keywords:** circSHPRH, circular RNA, proliferation, invasion, bladder cancer

## Abstract

Bladder cancer (BCa) is one of the most common tumors of the genitourinary system. However, the detailed molecular mechanism of BCa progression is still unclear. Recently, an increasing number of studies have demonstrated that circular RNAs (circRNAs) play a critical role in the tumorigenesis and progression of BCa. In this article, we showed that circSHPRH expression was obviously decreased in BCa tissues, compared with adjacent normal tissues. Moreover, a low circSHPRH level was positively correlated with a high grade, a high pathological stage, lymphatic metastasis and an unfavorable prognosis for BCa patients. Cell function studies indicated that silencing circSHPRH dramatically increased the proliferation, migration and invasion of BCa cells. Animal experiments revealed that circSHPRH overexpression repressed tumor growth. Mechanistic studies demonstrated that circSHPRH could combine with miR-942 and serve as a sponge of miR-942, which targets BARX2 in BCa cells. Rescue experiments showed that suppression of miR-942 or BARX2 overexpression could significantly abrogate the promoting effects of circSHPRH silencing on BCa cell proliferation and invasion. Furthermore, circSHPRH overexpression partly eliminated the suppressive effects of miR-942 on BARX2 expression. In addition, circSHPRH knockdown promoted activation of the Wnt/β-catenin signaling pathway by regulating BARX2. Taken together, our findings indicate that circSHPRH serves as a sponge of miR-942 to inhibit BCa progression by upregulating BARX2 expression, thereby inhibiting the Wnt/β-catenin signaling pathway.

## INTRODUCTION

Bladder cancer (BCa) is one of the most aggressive and malignant tumors of the urinary system [1]. In 2018, approximately 81,910 people were diagnosed with BCa in America [2]. Although several methods such as surgery, chemotherapy, radiotherapy, and immunotherapy have been used to treat BCa, the patients’ prognosis is still poor, with an estimated 17,240 deaths in 2018 in the United States [2]. Thus, elucidating the mechanisms of BCa progression and identifying a novel potential therapeutic target for the treatment of BCa are essential.

To date, many noncoding RNAs (ncRNAs), such as miRNAs [3] and long noncoding RNAs [4], have been confirmed to be involved in BCa progression. Circular RNAs (circRNAs) are a special type of single-stranded endogenous ncRNAs, which have a closed circular structure [5]. CircRNAs were previously considered to be byproducts of splicing errors due to technological limitations [6, 7]. Recently, numerous studies have indicated that circRNAs are conserved, abundantly expressed and not easily degraded in mammalian cells [5, 8]. Accumulating studies have shown that circRNAs play an important role in the proliferation and metastasis of cancer. For example, we previously reported that circFNDC3B and circUBXN7 serve as miRNA sponges to inhibit BCa progression [9, 10].

**Figure 1:**
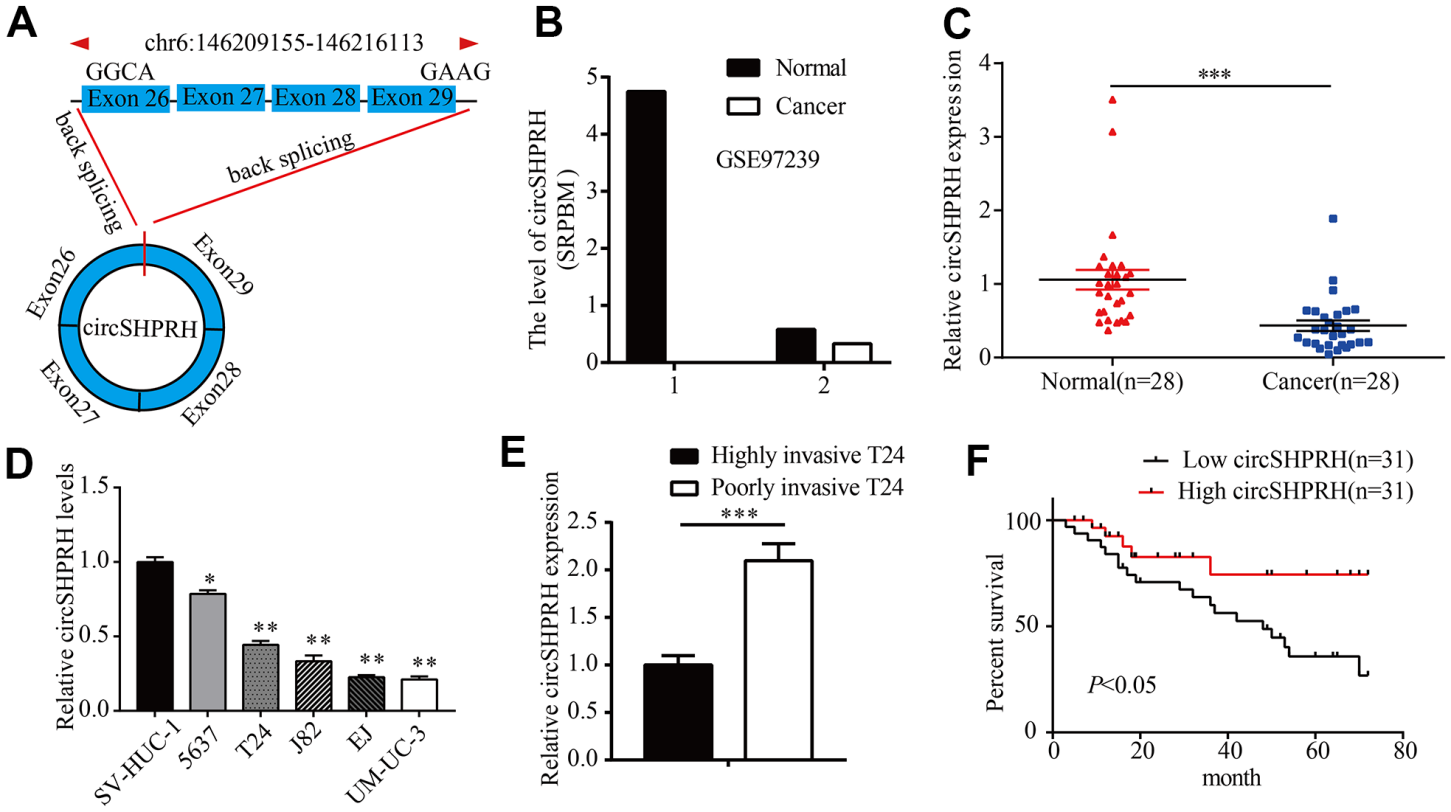
**circSHPRH is downregulated in BCa tissues and cell lines.** (**A**) circSHPRH was backspliced from exon 29 and exon 26 of the SHPRH gene. (**B**) The relative expression of circSHPRH in 2 BCa tissues and paired normal tissues according to the RNA-seq dataset (GSE97239). (**C**) The relative expression of circSHPRH in 28 BCa tissues and matched normal tissues, as detected by qRT–PCR. (**D**) The relative expression of circSHPRH in different BCa cell lines, as measured by qRT–PCR. (**E**) The relative expression of circSHPRH in highly invasive T24 and poorly invasive T24 cells. (**F**) Lower circSHPRH expression was associated with poorer survival, as determined by the Kaplan–Meier method. *P<0.05, **P<0.01, ***P<0.001.

Here, we analyzed the expression profile data of circRNAs for BCa from the GEO database (GSE97239), and identified circSHPRH (hsa_circ_0001649) as a dysregulated circRNA in BCa. Hsa_circ_0001649, which is backspliced from exon 29 and exon 26 of the SNF2 histone Linker PHD RING helicase (SHPRH) gene (Figure 1A), has been reported to serve as an anti-oncogene in different kinds of human tumors, such as glioblastoma [11], hepatocellular carcinoma [12], and pancreatic ductal adenocarcinoma [13]. Nevertheless, the definite role of circSHPRH in BCa is still unclear. In this article, we discussed the clinical significance, biological role and underlying mechanism of action of circSHPRH in BCa progression.

## RESULTS

### The expression of circSHPRH was downregulated in BCa and associated with patient survival

To study the clinical significance of circSHPRH in BCa patients, we first analyzed the RNA-Seq dataset (GSE97239) and found that the expression of circSHPRH in the two BCa tissues was lower than that in paired normal tissues (Figure 1B). We further detected circSHPRH expression in 28 pairs of BCa tissues and normal tissues. qRT–PCR results showed that circSHPRH expression in BCa tissues was significantly decreased compared with that in paired normal tissues (Figure 1C). Consistently, circSHPRH expression in the BCa cell lines was also downregulated compared with that in the immortalized uroepithelial cell line SV-HUC-1 (Figure 1D). We previously constructed a cell invasion model in BCa [9] and discovered that circSHPRH levels were also apparently decreased in highly invasive T24 cells compared with poorly invasive T24 cells (Figure 1E). Furthermore, we detected circSHPRH expression in a total of 62 BCa tissues. The chi-square test showed that BCa patients with a high grade, an advanced pathological T stage and positive lymph node metastasis had low circSHPRH levels. (Table 1). Subsequently, Kaplan-Meier analysis was performed to investigate the relationship between circSHPRH level and the prognosis of BCa patients. As shown in Figure 1F, BCa patients with lower circSHPRH expression had shorter survival times than those with higher circSHPRH expression, indicating that downregulated circSHPRH predicted a poor prognosis for BCa patients.

**Table 1:** Relationship between circSHPRH expression and clinicopathological characteristics.

**Characteristics**	**No.**	**circSHPRH expression**		***P*-value**
		**Low(n=31)**	**High(n=31)**	
Sex Male Female Age	47 15	24 7	23 8	0.767
<60	28	16	12	0.307
≥60	34	15	19	
T stage				
pT_a_-pT_1_	18	5	13	0.025*
pT_2_-T_4_	44	26	18	
Grade				
Low	20	6	14	0.030*
High	42	25	17	
Lymph node metastasis				
Yes	16	12	4	0.020*
No	46	19	27	

*P<0.05.

### Silencing circSHPRH promotes BCa cell proliferation, migration and invasion

**Figure 2:**
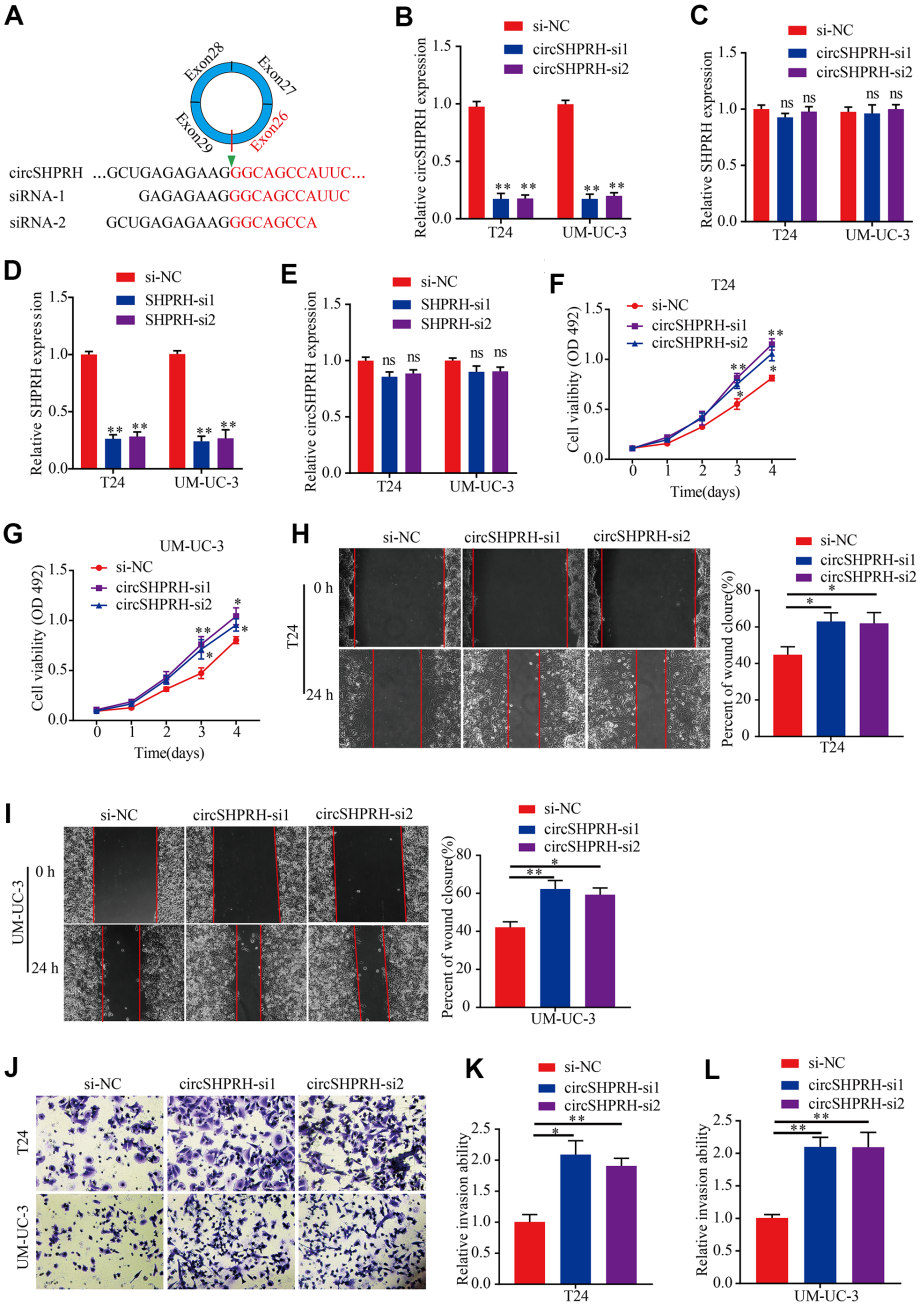
**circSHPRH knockdown promotes BCa cell proliferation, migration and invasion.** (**A**) si-circSHPRH was specifically designed to target the back-splice site of circSHPRH. (**B**, **C**) The relative expression of circSHPRH and SHPRH in T24 and UM-UC-3 cells transfected with si-NC or si-circSHPRH. (**D**, **E**) The relative expression of SHPRH and circSHPRH in T24 and UM-UC-3 cells transfected with si-NC or si-SHPRH. (**F**, **G**) circSHPRH knockdown enhanced cell viability in T24 and UM-UC-3 cells, as shown by the MTS assay. (**H**, **I**) circSHPRH knockdown promoted the migration ability of T24 and UM-UC-3 cells, as measured by wound healing assay. (**J**–**L**) circSHPRH knockdown promoted the invasion ability of T24 and UM-UC-3 cells, as detected by Transwell invasion assay. Magnification, 200×, *P<0.05, **P<0.01.

To gain insight into the function of circSHPRH in BCa, a circSHPRH silencing sequence was first designed to target the unique back-splice junction of circSHPRH (Figure 2A). Si-circSHPRH and si-NC were transiently transfected into BCa cells. qRT–PCR was conducted to verify the silencing efficiency, which showed that the abundance of circSHPRH was markedly downregulated after transfection (Figure 2B), but the SHPRH level was not dramatically affected (Figure 2C). Then, si-SHPRH was transfected into BCa cells (Figure 2D), and the data illustrated that compared with that in the si-NC group, circSHPRH expression was not notably affected in the si-SHPRH group (Figure 2E). Next, we conducted an MTS experiment to test the impact of circSHPRH on the proliferation capacity of BCa cells. The results showed that silencing circSHPRH obviously enhanced the cell proliferation ability (Figure 2F, 2G). Additionally, to assess the impacts of circSHPRH on BCa cell migration and invasion, we executed wound healing analysis and Transwell Matrigel invasion experiments. Our data suggested that circSHPRH knockdown dramatically increased the percentage of wound closure (Figure 2H, 2I) and the cell invasion ability (Figure 2J–2L). In conclusion, these results revealed that circSHPRH knockdown facilitated the malignant behaviors of BCa.

### circSHPRH directly binds to miR-942

**Figure 3:**
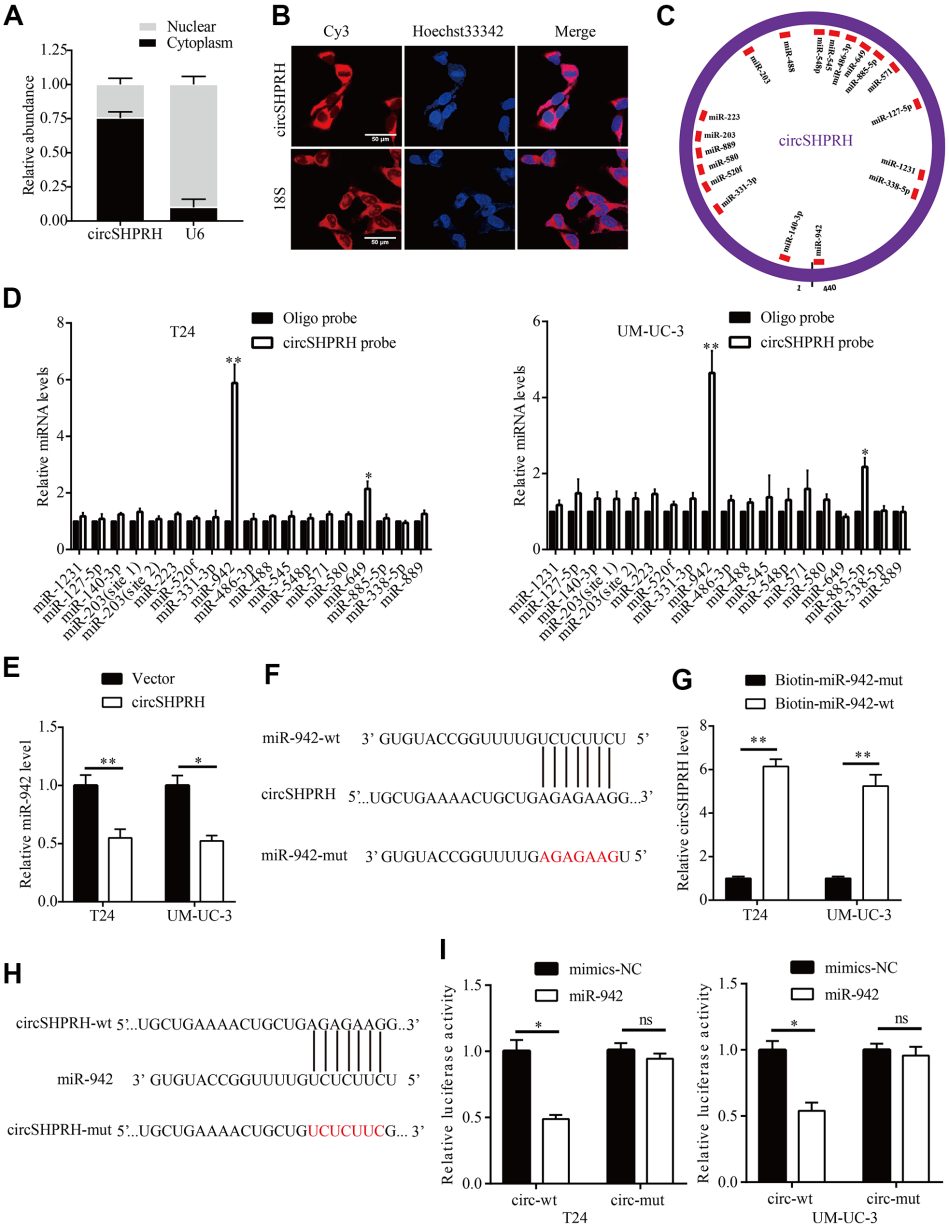
**circSHPRH directly binds to miR-942.** (**A**, **B**) circSHPRH was predominantly located in the cytoplasm of T24 cells, as detected by nucleus-plasma separation assay (**A**) and FISH (**B**). (**C**) Eighteen miRNAs possess potential circSHPRH binding sites, as predicted by CircInteractome. (**D**) miR-942 was pulled down and enriched with a circSHPRH specific probe in both T24 and UM-UC-3 cells, as detected by qRT–PCR. (**E**) The relative expression of miR-942 in T24 and UM-UC-3 cells transfected with the vector or circSHPRH plasmid. (**F**) The potential circSHPRH binding site and mutant sequence in miR-942. (**G**) circSHPRH was captured by miR-942 using a biotin-labeled miRNA capture assay. (**H**) The potential miR-942 binding site and mutant sequence in circSHPRH. (**I**) Luciferase activity was detected in T24 and UM-UC-3 cells transfected with wild-type (wt) circSHPRH (or mutant) and miR-942 mimics or mimics-NC. *P<0.05, **P<0.01.

To investigate the molecular mechanism of circSHPRH, nucleus-plasma separation experiments and FISH assays were performed to detect the subcellular localization of circSHPRH. The results revealed that circSHPRH was predominantly localized in the cytoplasm (Figure 3A, 3B). Then, we used CircInteractome to predict miRNAs that might potentially bind with circSHPRH, and the results demonstrated that 18 miRNAs (score>80) had potential circSHPRH binding sites (Figure 3C). We further performed an RNA pulldown assay to identify which miRNAs could bind to circSHPRH. The qRT–PCR results revealed that only miR-942 was abundantly detected in the fractions pulled down by the specific biotin-labeled circSHPRH probe in BCa cells (Figure 3D). In addition, circSHPRH overexpression significantly downregulated miR-942 levels in BCa cells (Figure 3E). Furthermore, circSHPRH was abundantly enriched in the RNA complex captured by wild-type biotin-labeled miR-942 (Figure 3F, 3G). To further demonstrate the interaction between circSHPRH and miR-942, a GP-miRGLO luciferase reporter plasmid containing wild-type circSHPRH harboring potential miR-942 binding sites (AGAGAAG) and a mutant reporter plasmid were constructed (Figure 3H). The dual luciferase reporter gene assays results showed that compared with that in the cotransfected mimics-NC and circSHPRH-wt vector group, the relative luciferase activity of the cotransfected miR-942 mimic and circSHPRH-wt vector group was significantly reduced. However, when we cotransfected mimics-NC or miR-942 mimics and circSHPRH-mut vector into BCa cells, the relative luciferase activity in the two groups was not significantly changed (Figure 3I).

### miR-942 acts as an oncogene and is sponged by circSHPRH

**Figure 4:**
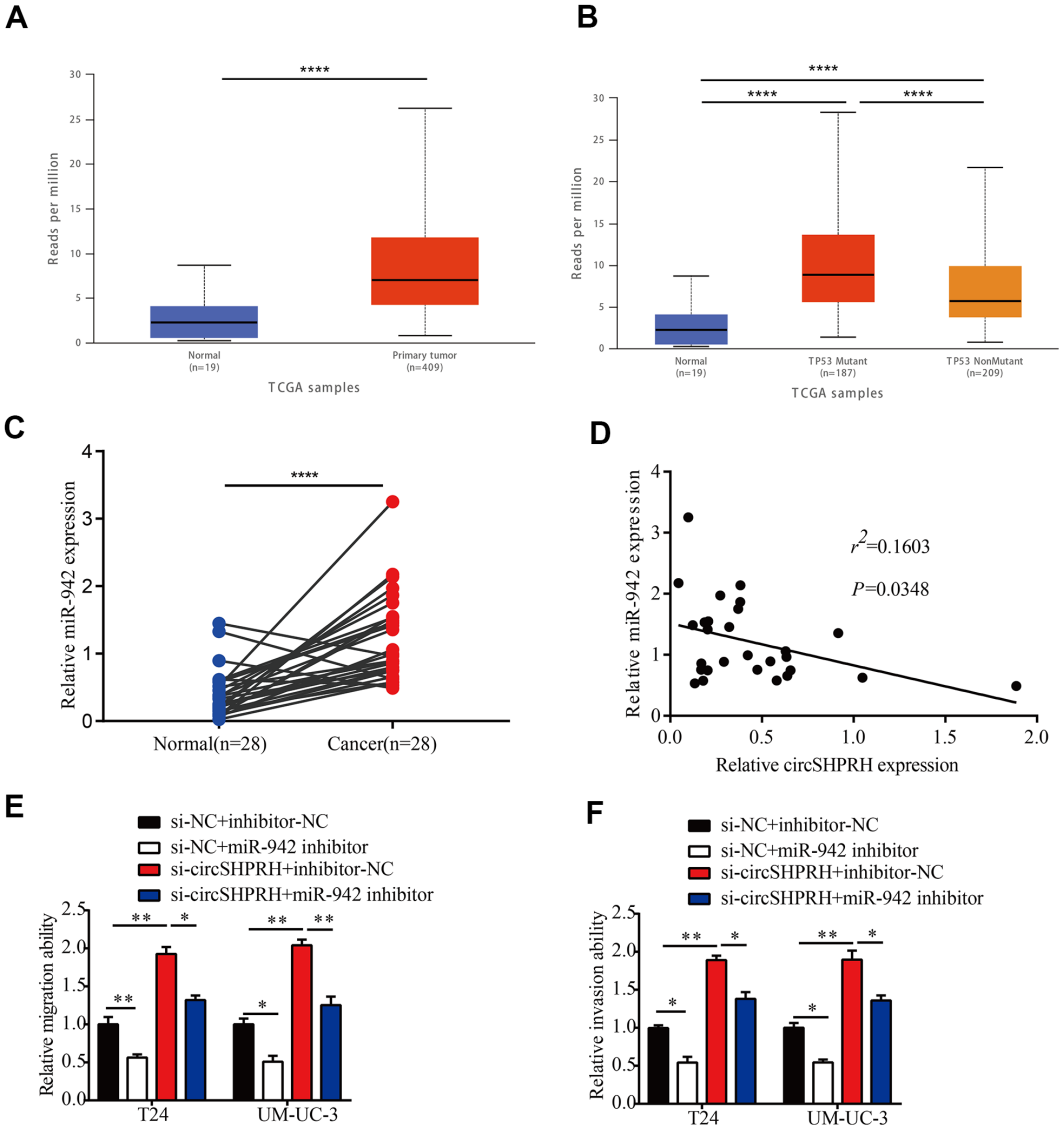
**miR-942 plays an oncogenic role and is sponged by circSHPRH in BCa.** (**A**) Comparison of miR-942 expression between BCa tissues and normal tissues from the TCGA dataset. (**B**) Expression of miR-942 in BCa based on TP53 mutation status analyzed by the UALCAN web server. (**C**) The relative expression of miR-942 in 28 BCa tissues and paired normal tissues, as measured by qRT–PCR. (**D**) Correlation between miR-942 and circSHPRH expression in BCa tissues by qRT–PCR. (**E**, **F**) Silencing miR-942 abrogated the cell migration (**E**) and invasion (**F**) abilities induced by circSHPRH knockdown.*P<0.05, **P<0.01, ****P<0.0001.

Numerous studies have confirmed that miR-942 plays an oncogenic role in diverse types of human malignancies [14–19]. However, the clinical significance of miR-942 is still unclear. Therefore, we first used UALCAN (http://ualcan.path.uab.edu) to analyze the expression of miR-942 in BCa tissues and normal tissues from the TCGA dataset. The results showed that the expression of miR-942 in primary tumor tissues (n=409) was higher than that in normal tissues (n=19) (Figure 4A). Moreover, TP53 mutant BCa tissues (n=187) exhibited higher miR-942 expression than TP53 nonmutant BCa tissues (n=209) (Figure 4B). Next, qRT–PCR was performed to analyze miR-942 levels in 28 BCa tissues and matched paracancerous tissues. The results revealed that the miR-942 level was notably upregulated in BCa tissues compared with paracancerous tissues (Figure 4C). Further correlation analysis showed that the relative miR-942 expression levels exhibited negative correlations with circSHPRH levels in 28 BCa tissues (Figure 4D). Moreover, rescue experiments revealed that suppression of miR-942 markedly diminished the migration and invasion capacities of BCa cells induced by circSHPRH knockdown (Figure 4E, 4F). Overall, these data demonstrated that miR-942 played an oncogenic role and was sponged by circSHPRH in BCa.

### miR-942 directly targets BARX2

**Figure 5:**
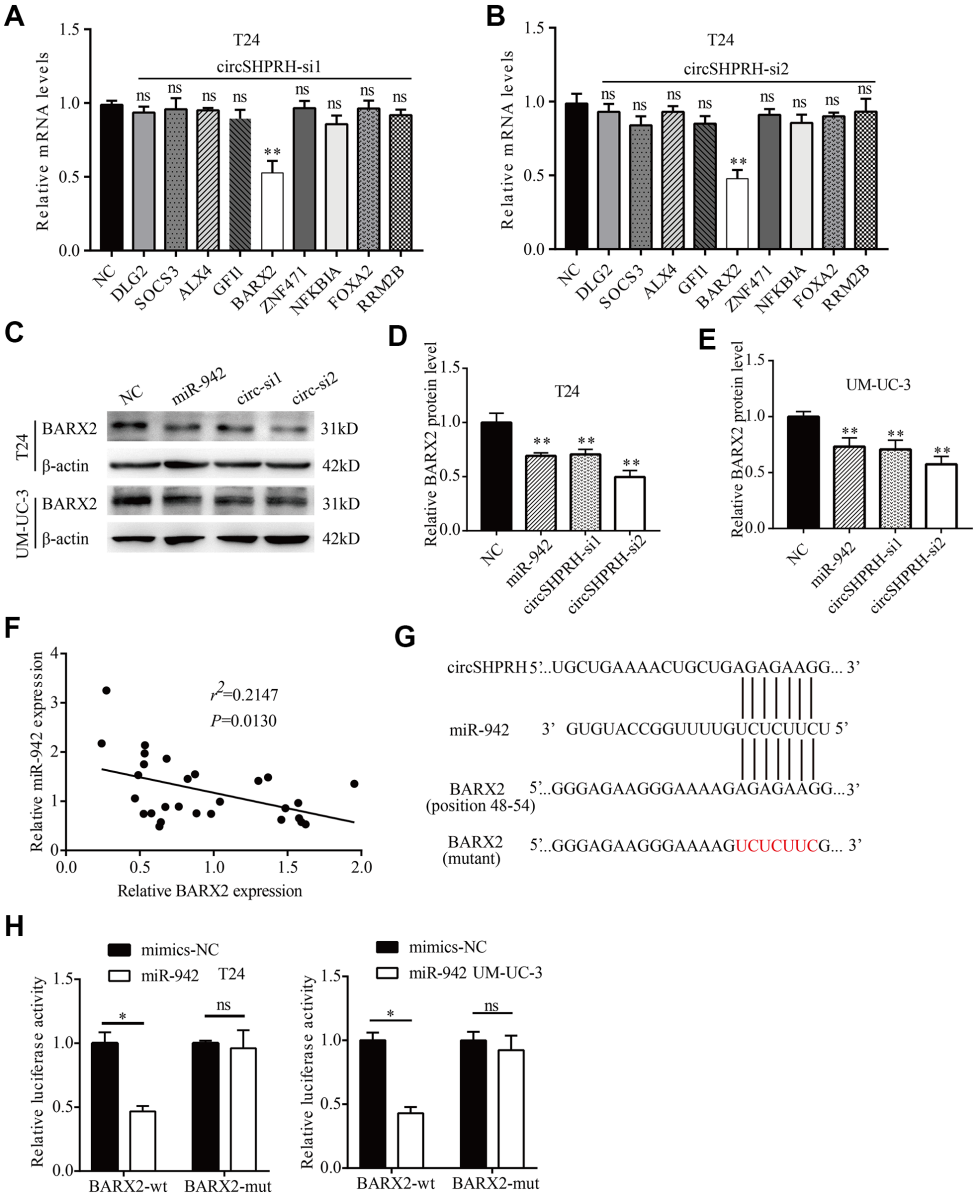
**miR-942 directly targets BARX2.** (**A**, **B**) The relative mRNA levels of 9 target genes of miR-942 in T24 cells transfected with si-NC or si-circSHPRH. (**C**–**E**) The effect of miR-942 overexpression or circSHPRH silencing on BARX2 expression in BCa cells, as detected by western blot. (**F**) Correlation between miR-942 and BARX2 mRNA expression in BCa tissues. (**G**) The putative sequences by which miR-942 bound to circSHPRH and the 3’-UTR of BARX2, as predicted by CircInteractome and TargetScan. (**H**) Luciferase activity was detected in T24 and UM-UC-3 cells transfected with the wild-type (wt) BARX2-3’-UTR (or mutant) and miR-942 mimics or mimics-NC.

To further identify the downstream target of circSHPRH/miR-942, we first screened 9 tumor suppressors (DLG2, SOCS3, ALX4, GFI1, BARX2, ZNF471, NFKBIA, FOXA2, RRM2B) that were confirmed to be the direct targets of miR-942 in published papers [15, 17–24]. Then, we investigated whether circSHPRH silencing could influence these genes at the mRNA level. The results revealed that circSHPRH silencing downregulated only BARX2 expression, while the levels of the other genes were not influenced in T24 cells (Figure 5A, 5B). We therefore chose BARX2 for further study. Western blot analysis showed that silencing circSHPRH significantly downregulated BARX2 expression at the protein level in BCa cells (Figure 5C–5E). BARX2, a homeobox gene of the Bar class, is involved in developmental processes. For example, BARX2 regulates chondrogenesis during limb development [25] and is essential for muscle growth and regeneration [26]. However, BARX2 has been demonstrated to serve as an anti-oncogene in several malignant tumors, including breast cancer [27] and ovarian cancer [28]. To evaluate the function of BARX2 in BCa, we executed qRT–PCR to investigate BARX2 levels in 28 BCa tissues. Correlation analysis showed that the relative level of BARX2 was negatively related to miR-942 expression (Figure 5F). Additionally, when miR-942 mimics were transfected into BCa cells, BARX2 protein levels were significantly downregulated compared with those in the NC group (Figure 5C–5E). To confirm that miR-942 directly targets the 3’UTR of BARX2, GP-miRGLO plasmids containing the 3’UTR of wild-type or mutant BARX2 were constructed (Figure 5G). Subsequently, dual luciferase reporter gene experiments were performed to verify whether miR-942 could directly combine with the BARX2 3’UTR. Our data demonstrated that miR-942 overexpression distinctly decreased the relative luciferase activity of the wild-type BARX2-3’UTR, and no significant luciferase activity was observed once the putative binding sites of the BARX2-3’UTR were mutated (Figure 5H). These data showed that BARX2 served as a direct target of miR-942.

### miR-942 promotes BCa cell proliferation by targeting BARX2

**Figure 6:**
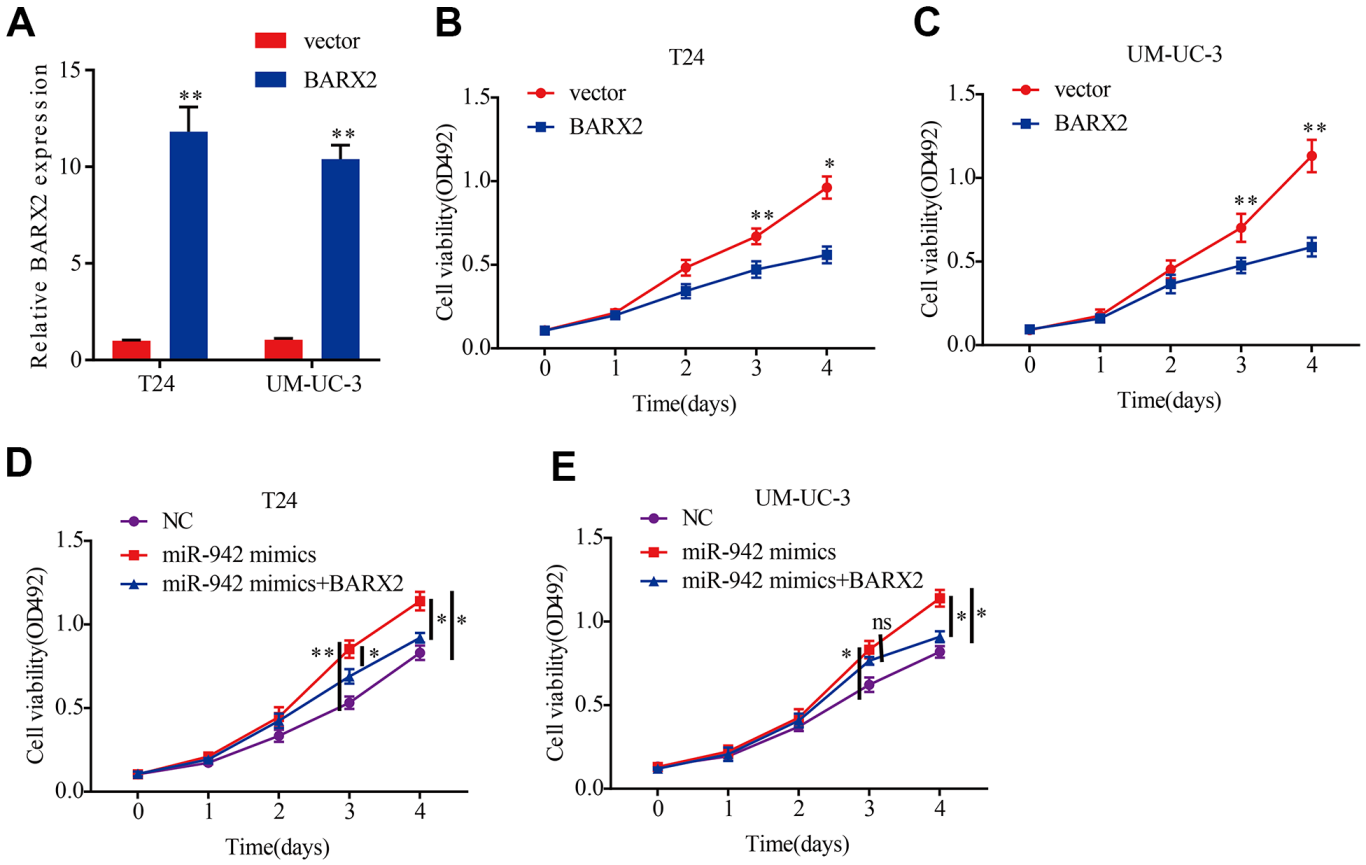
**miR-942 promotes the proliferation abilities of BCa cells by targeting BARX2.** (**A**) Relative expression of BARX2 in T24 and UM-UC-3 cells transfected with vector or BARX2 plasmid. (**B**, **C**) BARX2 overexpression inhibited cell viability in T24 and UM-UC-3 cells, as shown by the MTS assay. (**D**, **E**) Overexpression of BARX2 partially abrogated the cell proliferation abilities induced by miR-942 overexpression.*P<0.05, **P<0.01.

To clarify whether miR-942 enhances cell proliferation abilities by targeting BARX2, we first investigated the molecular function of BARX2. Then, we transfected the BARX2 overexpression vector into BCa cells and qRT–PCR was carried out to verify the transfection efficiency (Figure 6A). Next, we performed MTS experiments to investigate cell proliferation abilities. Our data suggested that upregulation of BARX2 dramatically attenuated the proliferation capacities of BCa cells (Figure 6B, 6C). The above findings indicated that BARX2 was also a tumor suppressor in BCa. Then, a rescue experiment was performed to explore whether miR-942 facilitates the proliferation ability of BCa cells by regulating BARX2. MTS experiments revealed that upregulation of miR-942 dramatically increased the viability of T24 and UM-UC-3 cells. However, these impacts were partially mitigated by overexpression of BARX2 (Figure 6D, 6E).

### circSHPRH inhibits BCa cell proliferation and invasion through the miR-942/BARX2 pathway

**Figure 7:**
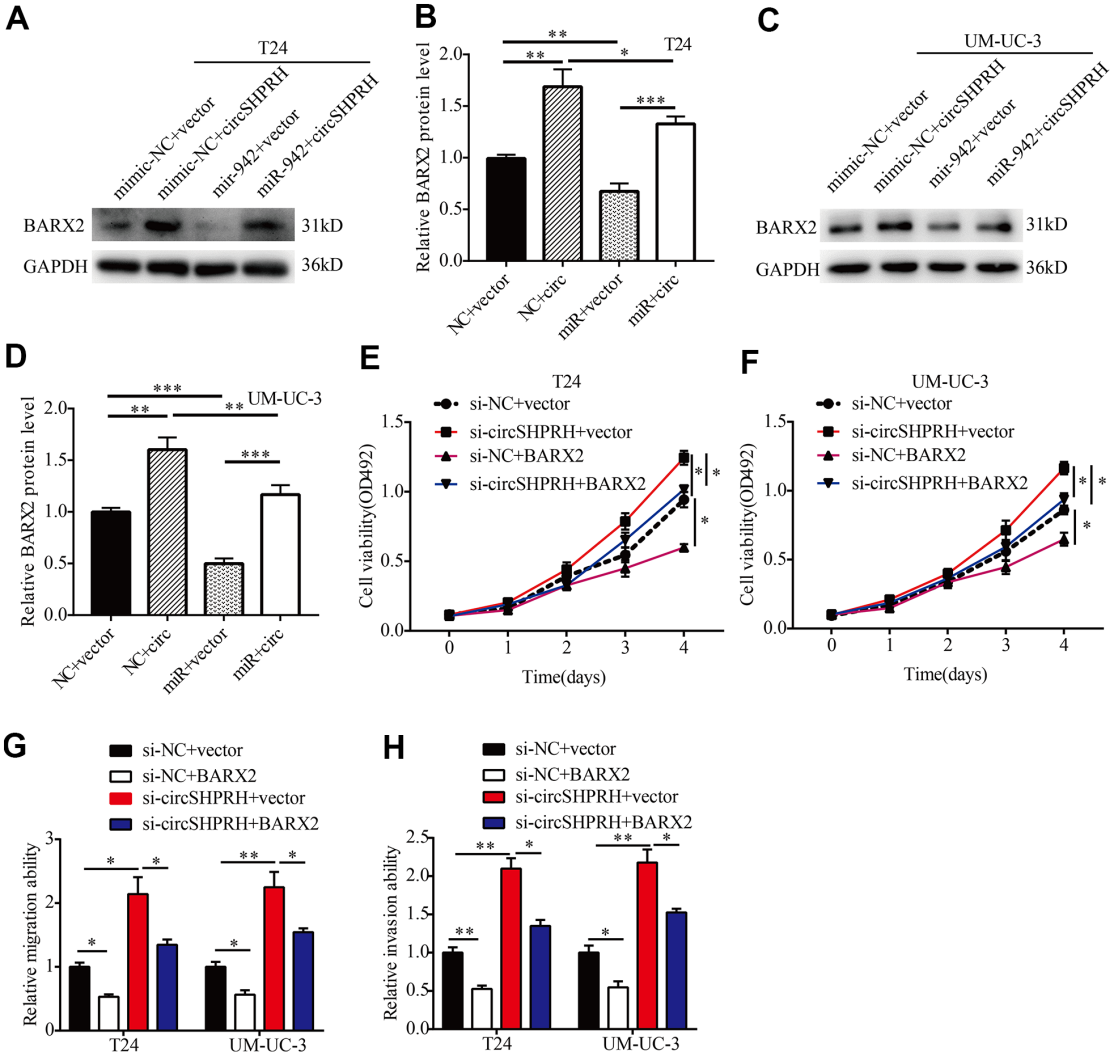
**circSHPRH suppresses the malignant behavior of BCa through the miR-942/BARX2 pathway.** (**A**–**D**) Rescue assays indicated that the inhibitory effect of miR-942 on BARX2 was partially reversed by circSHPRH overexpression in T24 (**A**, **B**) and UM-UC-3 cells (**C**, **D**). (**E**–**H**) Rescue assays indicated that the promoting effect of circSHPRH knockdown on cell proliferation (**E**, **F**), migration (**G**) and invasion (**H**) in T24 and UM-UC-3 cells was abrogated by BARX2 overexpression. *P<0.05, **P<0.01.

To further investigate the impacts of circSHPRH on the miR-942/BARX2 pathway, a rescue experiment was performed. Western blot analysis showed that circSHPRH overexpression significantly upregulated BARX2 expression while miR-942 overexpression abrogated the promoting effect of circSHPRH on BARX2 (Figure 7A–7D), indicating that circSHPRH enhanced BARX2 expression by sponging miR-942. Next, we evaluated whether BARX2 played a vital role in circSHPRH mediated BCa cell malignant behavior. Rescue experiments indicated that circSHPRH knockdown enhanced BCa cell proliferation, migration and invasion, and these impacts were partially abrogated via BARX2 overexpression (Figure 7E–7H), demonstrating that BARX2 served as an anti-oncogene in BCa and had a vital role in the circSHPRH/miR-942/BARX2 axis.

### circSHPRH knockdown activates the Wnt/β-catenin signaling pathway by regulating BARX2

**Figure 8:**
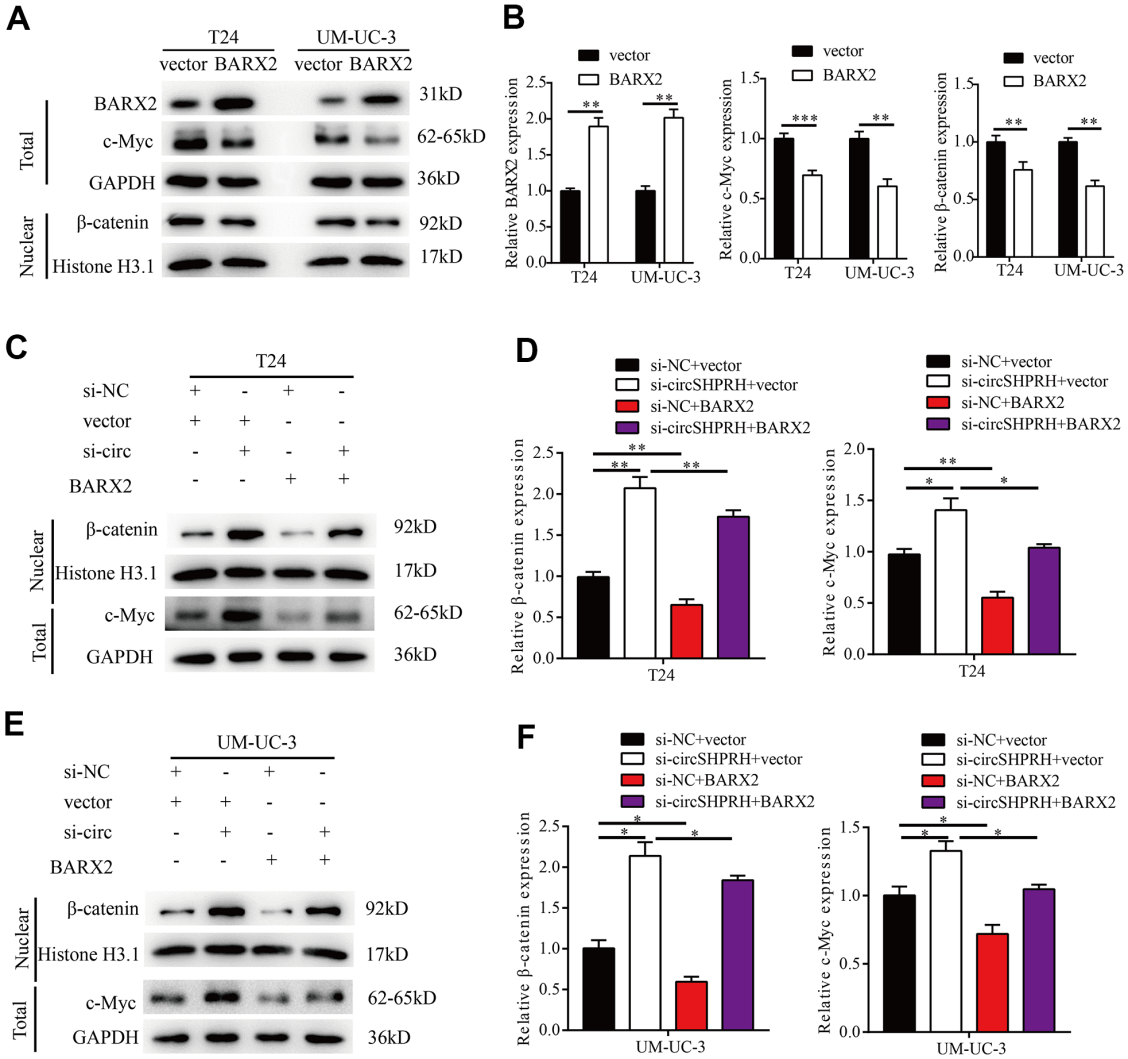
**circSHPRH knockdown activates the Wnt/β-catenin signaling pathway by regulating BARX2.** (**A**, **B**) Relative expression of BARX2, β-catenin and c-MYC in T24 and UM-UC-3 cells transfected with vector or BARX2 plasmid. (**C**–**F**) Rescue assays indicated that the promoting effect of circSHPRH knockdown on c-MYC and nuclear β-catenin expression was partially diminished by BARX2 overexpression in T24 (**C**, **D**) and UM-UC-3 cells (**E**, **F**).*P<0.05, **P<0.01.

BARX2 has been previously reported to be a negative regulator in the classic Wnt/β-catenin pathway [29–31]. Western blotting was then performed to study whether the Wnt/β-catenin signaling pathway could be regulated through BARX2 in BCa. In our study, BARX2 overexpression markedly decreased c-MYC and nuclear β-catenin expression (Figure 8A, 8B). Furthermore, we found that circSHPRH knockdown increased the expression of c-MYC and nuclear β-catenin, and these effects were partially abrogated by overexpression of BARX2 in BCa cells (Figure 8C–8F).

### circSHPRH overexpression inhibits BCa growth *in vivo*

**Figure 9:**
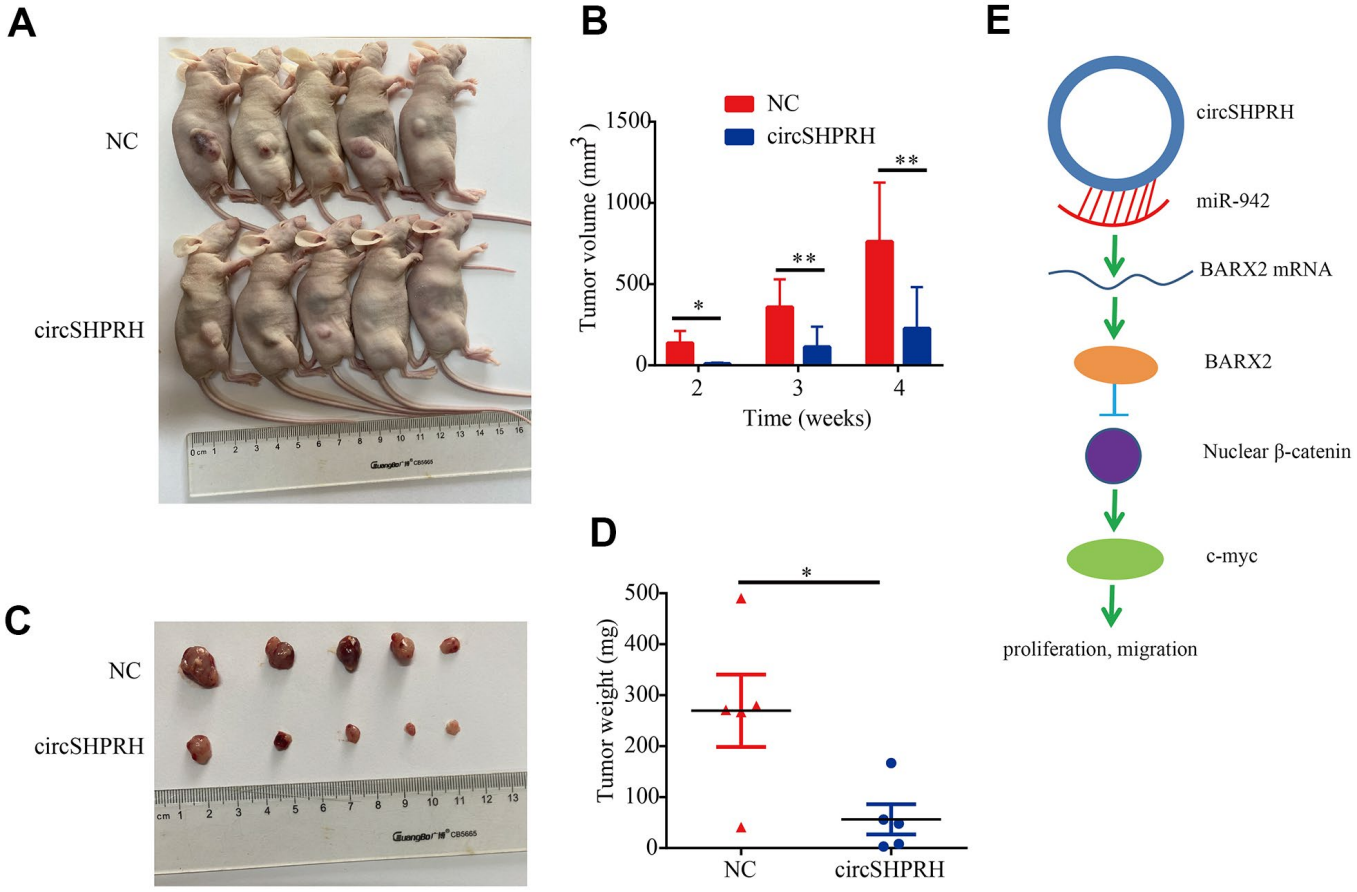
**Overexpression of circSHPRH represses tumor growth *in vivo*.** (**A**) Representative images of subcutaneous tumors in BALB/c nude mice. (**B**) The tumor volume of xenograft tumors at different time points. (**C**) Representative images of dissected tumors from sacrificed mice. (**D**) Compared with the control group, the tumor weights significantly decreased in the circSHPRH-overexpressing group. (**E**) Schematic diagram shows that circSHPRH inhibits BCa cells proliferation and migration through the miR-942/BARX2/Wnt/β-catenin signaling pathway.

We investigated the biological role of circSHPRH on the growth of BCa *in vivo*, UM-UC-3 cells with stable circSHPRH overexpression and negative control UM-UC-3 cells with lentivirus transfection were constructed. Then, the flanks of BALB/c nude mice were subcutaneously injected with cells in the circSHPRH-overexpressing and corresponding NC groups (Figure 9A). The longest diameter and shortest diameter of the palpable tumors were measured weekly. We found that the volume of tumors from the circSHPRH-overexpressing group was obviously reduced compared with that in the NC group (Figure 9B, 9C). A distinct decrease in tumor weight was also discovered in the nude mice inoculated with circSHPRH-overexpressing cells (Figure 9D).

## DISCUSSION

For a long time in the past, circRNAs attracted considerable attention because of their vital roles in a variety of human diseases, including BCa. In the present study, we focused on a well-known tumor suppressor, circSHPRH, which is located at chr 6: 146209155-146216113. Nevertheless, the function of circSHPRH in BCa has not been clarified. Our research showed that circSHPRH expression was markedly decreased in BCa tissues and cell lines. Moreover, the expression of circSHPRH correlated with a high grade, an advanced pathological stage, and lymph node metastasis. Furthermore, BCa patients with lower circSHPRH expression had worse prognoses. Functional experiments confirmed that circSHPRH knockdown dramatically enhanced BCa cell proliferation, migration and invasion. In addition, overexpression of circSHPRH repressed tumor growth *in vivo*. These data demonstrated that circSHPRH serves as a tumor suppressor in BCa.

circSHPRH regulates tumor progression through different mechanisms in different kinds of human tumors. For example, circSHPRH can encode a novel protein called SHPRH-146aa, which can suppress cell proliferation and tumorigenicity in glioma [32]. Xing L et al. indicated that downregulation of hsa_circ_0001649 promoted cell proliferation and inhibited apoptosis by regulating the AKT/mTOR signaling pathway [33]. Numerous studies have pointed out that exonic circRNAs serve as miRNA sponges to modulate targeted gene expression in tumorigenesis [12]. To clearly elucidate the mechanism of circSHPRH in BCa, we preliminarily detected its subcellular location by nucleus-plasma separation and FISH assays, and the results revealed that circSHPRH was mostly found in the cytoplasm. Further bioinformatics analysis revealed that circSHPRH contains miR-942 binding sites. RNA pulldown assays validated that circSHPRH directly interacted with miR-942. Moreover, biotin-labeled miR-942 captured abundant circSHPRH in BCa cells. miR-942 has been demonstrated to play a carcinogenic role in gastric cancer [15], breast cancer [18], hepatocellular carcinoma [19], colorectal cancer [14], non-small-cell lung cancer [17] and ovarian cancer [16]. In this work, based on TCGA samples and our own clinical samples, we discovered that circSHPRH was downregulated in BCa tissues relative to normal tissues. Moreover, functional experiments indicated that inhibition of miR-942 considerably weakened the migration and invasion capacities of BCa cells. In BCa tissues, miR-942 expression was inversely linked with circSHPRH expression. In addition, rescue experiments indicated that circSHPRH knockdown partially eliminated the tumor-inhibitory impact of miR-942 silencing on BCa cell migration and invasion. Our studies confirmed that circSHPRH suppresses BCa progression by sponging miR-942.

We further studied the direct target gene of the circSHPRH/miR-942 axis by western blot and dual luciferase reporter gene assays. BARX2 has been demonstrated to be the downstream target of miR-942, which is consistent with Yang’s study [17]. BARX2 plays a negative role in regulating the canonical Wnt/β-catenin pathway [29–31] and serves as an anti-oncogene in non-small-cell lung cancer [17, 29], colorectal cancer [34], hepatocellular carcinoma [35], breast cancer [27] and ovarian cancer [28]. However, the functions of BARX2 in BCa have not been illustrated. Our results demonstrated that the viability and invasion of BCa cells were significantly repressed by BARX2 overexpression. Moreover, BARX2 expression was upregulated by circSHPRH overexpression and downregulated by miR-942 overexpression. Further rescue experiments revealed that circSHPRH upregulated BARX2 expression by sponging miR-942. In addition, BARX2 partially abrogated the tumor-promoting impacts of circSHPRH knockdown on BCa cell proliferation, migration and invasion. A subsequent study confirmed that circSHPRH knockdown activates the Wnt/β-catenin signaling pathway by regulating BARX2. According to our findings, the circSHPRH/miR-942/BARX2/Wnt/β-catenin axis might play a pivotal role in BCa progression (Figure 9E).

In conclusion, this study reveals a tumor-suppressing role of circSHPRH in BCa progression by sponging miR-942 and upregulating BARX2 expression. Our studies provide novel insights into BCa progression and offer a new potential therapeutic target for BCa treatment.

## MATERIALS AND METHODS

### Patient samples

Primary BCa tissues and paired paracancerous tissues were collected from patients who underwent radical cystectomy at Sun Yat-Sen Memorial Hospital, Sun Yat-Sen University. Total tissues were pathologically and histologically confirmed by three pathologists. The clinical and pathological characteristics of all BCa patients are shown in Table 1. According to the ethical guidelines of the Declaration of Helsinki, we developed an experimental protocol. Then, the research was authorized by the Human Ethics Committee of Sun Yat-Sen Memorial Hospital.

### Cell culture and transfection

Human BCa cell lines (5637, T24, J82, EJ, and UM-UC-3 cells) and the human immortalized uroepithelium cell line SV-HUC-1 were acquired from the American Type Culture Collection. RPMI medium 1640 (Gibco, USA) or DMEM (Gibco) mixed with 10% fetal bovine serum (BI, Israel) and 1% penicillin/streptomycin (Gibco) was used to cultivate BCa cell lines. F-12K medium (Gibco, USA) was used to cultivate SV-HUC-1 cells. All cell lines were grown at 37° C in a humidified environment containing 5% CO_2_.

miR-942 mimics, miR-942 inhibitor, si-circSHPRH and the corresponding controls (GenePharma, China) were transfected into BCa cells using Lipofectamine RNAimax (Invitrogen, USA). The plenti-ciR-GFP-T2A vector (IGE Biotech Co, China) was used for circSHPRH overexpression, and pcDNA 3.1 (Addgene, USA) was used for BARX2 cloning. BCa cells were transiently transfected with the BARX2 overexpression plasmid using X-treme (Sigma, USA).

### Isolation of highly invasive and poorly invasive T24 cells

Highly invasive and poorly invasive T24 cells were isolated by repeated Transwell Matrigel invasion assays. As we previously described [9], T24 cells were starved and cultured with serum medium for 24 h. Subsequently, 1 mL serum-free culture medium containing 5×10^5^ cells was plated in the upper chamber of six-well Transwell plates (Corning, USA), which were precoated with Matrigel (BD Biosciences, USA), and 2.5 mL of RPMI-1640 medium containing 20% FBS was added to the lower well. After 24 h of incubation, the highly invasive T24 cells in the lower chamber and the poorly invasive T24 cells in the upper chamber of the six-well Transwell plates were harvested under sterile conditions. After ten screenings, cells that did not penetrate the membrane were considered poorly invasive T24 cells, while the cells that successfully penetrated the membrane were identified as highly invasive T24 cells.

### RNA isolation and qRT–PCR

RNAiso Plus (Takara, Japan) was used to isolate total RNA from cells and tissues. PrimeScript RT Master Mix (TaKaRa, Japan) was used to synthesize circRNA and mRNA into cDNA. According to the instructions, an miRNA First-Strand cDNA Synthesis Kit (Sangon Biotech, China) was used to synthesize miRNA into cDNA. Then, qPCR was conducted using SYBRGreen Master Mix (Takara, Japan) on a Quantstudio™ DX system (Applied Biosystems, Singapore). The quantification of circRNA and mRNA expression was normalized to that of GAPDH, and U6 was used as the control for miRNA expression.

### MTS assay

After 24 h of transfection, 1,000 cells were plated into a transparent 96-well plate, and MTS assays (Promega, USA) were executed to assess cell viability. The absorbance values were measured at a wavelength of 492 nm.

### Wound healing assay

First, BCa cells were separately seeded into 6-well plates. After incubation for 24 h, a straight scratch was drawn with a 200 μL pipette tip. Images of the wounds were captured with an inverted microscope (Olympus, Japan) at the same position at 0 h and 24 h. Three random fields were selected to calculate the percentage of wound closure.

### Cell invasion assay

For the Transwell invasion assay, 200 μL serum-free culture medium containing 2×10^5^ cells were plated into the upper chamber of a 24-well plate, which was precoated with Matrigel (BD Biosciences, USA). Then, 600 μL medium containing 10% FBS was added to the lower chamber. After incubation for 24 h, the cells that migrated to the membrane of the upper chamber were fixed with 4% paraformaldehyde and stained with 1% crystal violet. The images were photographed and counted under an inverted microscope (Olympus, Japan) in three random fields.

### Nucleus-plasma separation assay

Cytoplasmic and nuclear RNA were separated using a PARIS™ kit (Invitrogen, USA). Briefly, 500 μL ice-cold Cell Fraction Buffer was used to lyse the T24 cells (1×10^7^) for 10 min. After centrifugation for 5 min, the supernatant containing cytoplasm was carefully transferred to a new centrifuge tube. In addition, the nuclear pellet was resuspended in 500 μL ice-cold Cell Disruption Buffer. Subsequently, the suspension with the cytoplasmic lysate and nuclear fraction were suspended in 500 μL of 2×Lysis/Binding Solution to isolate the RNA. NE-PER Nuclear and Cytoplasmic Extraction Reagents (Thermo Scientific, USA) were used to isolate nuclear and cytoplasmic proteins according to the manufacturer's recommendations.

### Fluorescence *in situ* hybridization (FISH) assay

According to the instructions, a Fluorescence *in Situ* Hybridization Kit (GenePharma, China) was used for FISH assays. Briefly, T24 cells were first plated in a confocal culture dish, and when the cells grew to 80% of the surface area of the dish, the cells were fixed with 0.5% paraformaldehyde for 30 min, prehybridized with hybridization buffer for 2 h at 37° C, and hybridized with a Cy3-labeled circSHPRH probe (GenePharma, China) in hybridization buffer [9, 36] at 37° C overnight. Then, the nucleus was restained with Hoechst 33342. A confocal microscope (LSM800, Carl Zeiss AG, Germany) was used to capture images.

### Prediction of circRNA-miRNA associations

In this study, CircInteractome (https://circinteractome.irp.nia.nih.gov/) was used to predict circSHPRH-miRNA interactions.

### RNA pull down assay and miRNA capture assay

Biotin-labeled RNA pulldown assays and miRNA capture assays were performed according to our reported method [9]. The biotinylated circSHPRH probe, biotin-labeled miR-942 mimic and negative control were obtained from GenePharma, Suzhou, China.

### Dual luciferase reporter gene assays

A sequence with miR-942 potential binding sites was designed from circSHPRH. The sequence was inserted into the GP-miRGLO plasmid (GenePharma, Shanghai, China), and then confirmed for correct insertion by sequencing. To detect the specificity of the binding site, the sequences that combined with the miR-942 seed sequence were mutated (from AGAGAAG to TCTCTTC), and the mutant sequence was inserted into the GP-miRGLO plasmid luciferase reporter. A fragment of the BARX2 3’-UTR containing the miR-942 binding site was inserted into the GP-miRGLO plasmid. The sequences that combined with the miR-942 seed sequence were mutated (from AGAGAAG to TCTCTTC), and the mutant BARX2 3’-UTR was inserted into the GP-miRGLO plasmid. For the dual luciferase reporter gene assays, the cells were seeded into a 24-well plate at a density of 3 × 10^4^ cells per well. After 24 h, the cells were transfected with luciferase plasmids or miRNA mimics (GenePharma, China) for 48 h. Then, a dual luciferase reporter gene experiment detection system (Promega, USA) was used to detect Renilla luciferase activity, and Renilla luciferase reporter activity was normalized with respect to firefly luciferase.

### Western blotting

A mixture of RIPA lysis buffer (Beyotime, China) and protease inhibitor (CMBIO, China) at a ratio of 100:1 was used to lyse BCa cells. Samples containing 30 μg of total protein were separated by 10% SDS–PAGE, and then the protein was transferred onto PVDF membranes for 2 hours. After blocking with 5% nonfat milk in TBST for 1 h at room temperature, anti-BARX2 (Bioss, China; cat. No. bs-19273R-3; dilution: 1:800), anti-c-MYC (Proteintech, China; cat. NO. 10828-1-AP; dilution: 1:5000), anti-β-catenin (Proteintech, China; cat. NO. 51067-2-AP; dilution: 1:5000), anti-GAPDH (Proteintech, China; cat. NO. 10494-1-AP; dilution: 1:10000), anti-Histone H3.1 (Beijing Ray Antibody Biotech, China; cat. NO. RM2005L; dilution 1:5000) and anti-β-actin (Proteintech, China; cat. No. 66009-1-Ig; dilution: 1:10000) antibodies were used to incubate PVDF membranes at 4° C overnight. The next day, the membranes were incubated with HRP-coagulated anti-rabbit or anti-mouse antibody at room temperature for 1 h.

### Tumor xenograft

For tumor xenograft assays, we randomly selected 10 female BALB/c nude mice (4 weeks old) and divided them into two groups. Stably circSHPRH-overexpressing and negative control UM-UC-3 cells were inoculated subcutaneously into the flank of each mouse (5 × 10^6^ cells per mouse). The longest diameter and shortest diameter of the palpable tumors were measured weekly. Four weeks after injection, the mice were sacrificed to assess the dissected tumor weight.

### Sequences of primers and oligonucleotides

The sequences of the primers and oligonucleotides used in this study are listed in Supplementary Table 1.

### Statistical analysis

The results are presented as the mean±SD of three independent experiments. SPSS 17.0 software (SPSS Inc., Chicago, IL, USA) or GraphPad Prism 7 software (GraphPad Software, Inc., La Jolla, CA, USA) was used to analyze the data. The chi-square test was conducted to analyze the correlation of circSHPRH expression with clinicopathological characteristics. Two-tailed Student’s t test was utilized to analyze differences between two groups. Overall survival analysis was performed using the Kaplan–Meier method and the log-rank test. *P*<0.05 was considered statistically significant.

## Supplementary Material

Supplementary Table 1
