# Supplementary material for: Circular RNA circSHPRH inhibits the malignant behaviors of bladder cancer by regulating the miR-942/BARX2 pathway
Source: Aging (Albany NY). 2022 Feb 24;14(4):1891–909. doi: 10.18632/aging.203911 (PMC8908925; doi:10.18632/aging.203911)
Supplement: Supplementary Table 1 [file aging-14-203911-s001.pdf]

SUPPLEMENTARY TABLE

Supplementary Table 1. The sequences of primers and oligonucleotides used in this study.

Primers	
circSHPRH-F	AGGCAATGCTGAAAACTGCT
circSHPRH-R	GCCACGTTGAGAAAACGAGT
SHPRH-F	AGATGATCCGGAGAGTGAGC
SHPRH-R	ACAGCCTCTCTTTGGTAGGG
DLG2-F	ACGCTCCACATGATCATTCC
DLG2-R	TTATAGGAGCAGGACTGGCC
SOCS3-F	GTCATTGGAGAGGCTGGACT
SOCS3-R	CTGTCCAGCCCAATACCTGA
GFI1-F	GGACCAGACTATTCCCTCCG
GFI1-R	AGTCCTCAAACTCCGAGCTC
BARX2-F	AAGCACGTGATGTCCCCTTA
BARX2-R	GTTTACTGGGCTGCTGGAAG
ZNF471-F	CCCTCCCAAGACACTGTTCT
ZNF471-R	TCACTCGTCATCTCCCAAGG
NFKBIA-F	GTCAAGGAGCTGCAGGAGAT
NFKBIA-R	CTTCACCTGGCGGATCACTT
FOXA2-F	ACTTCCAACTACCGCCTCC
FOXA2-R	ACAAACGACCAGCAATCACC
RRM2B-F	CCAGCCCGTTAGATTGCAAG
RRM2B-R	CCCAAAGTCAGCTCCTTCCT
ALX4-F	GTTCGGCACAACTTTCCTGT
ALX4-R	CTGGGGCTGGAACTTGTTAA
GAPDH-F	GGAGCGAGATCCCTCCAAAAT
GAPDH-R	GGCTGTTGTCATACTTCTCATGG
miR-1231	GTGTCTGGGCGGACAGCTGC
miR-127-5p	CTGAAGCTCAGAGGGCTCTGAT
miR-140-3p	TACCACAGGGTAGAACCACGG
miR-203	GTGAAATGTTTAGGACCACTAG
miR-223	TGTCAGTTTGTCAAATACCCCA
miR-520f	AAGTGCTTCCTTTTAGAGGGTT
miR-331-3p	GCCCCTGGGCCTATCCTAGAA
miR-942	TCTTCTCTGTTTTGGCCATGTG
miR-486-3p	CGGGGCAGCTCAGTACAGGAT
miR-488	TTGAAAGGCTATTTCTTGGTC
miR-545	TCAGCAAACATTTATTGTGTGC
miR-548p	TAGCAAAAACTGCAGTTACTTT
miR-571	TGAGTTGGCCATCTGAGTGAG
miR-580	TTGAGAATGATGAATCATTAGG
miR-649	AAACCTGTGTTGTTCAAGAGTC
miR-885-5p	TCCATTACACTACCCTGCCTCT
miR-338-5p	AACAATATCCTGGTGCTGAGTG
miR-889	AATGGCTGTCCGTAGTATGGTC
U6	CTCGCTTCGGCAGCACA

miR reverse	Sangon Biotech, China
**siRNAs**	
si-NC sense	UUCUCCGAACGUGUCACGUTT
si-NC antisense	ACGUGACACGUUCGGAGAATT
si-circSHPRH-1 sense	GAGAGAAGGGCAGCCAUUCTT
si-circSHPRH-1 antisense	GAAUGGCUGCCCUUCUCUCTT
si-circSHPRH-2 sense	GCUGAGAGAAGGGCAGCCATT
si-circSHPRH-2 antisense	UGGCUGCCCUUCUCUCAGCTT
si-SHPRH-1 sense	GCGUUUGAGUGGGAUUAAUTT
si-SHPRH-1 antisense	AUUAAUCCCACUCAAACGCTT
si-SHPRH-2 sense	GCGAGUGCGAAAUGAAAUATT
si-SHPRH-2 antisense	UAUUUCAUUUCGCACUCGCTT
**miR-942 mimics and inhibitors**	
mimics NC sense	UUCUCCGAACGUGUCACGUTT
mimics NC antisense	ACGUGACACGUUCGGAGAATT
miR-942 mimics sense	UCUUCUCUGUUUUGGCCAUGUG
miR-942 mimics antisense	CAUGGCCAAAACAGAGAAGAUU
inhibitor NC	CAGUACUUUUGUGUAGUACAA
miR-942 inhibitor	CACAUGGCCAAAACAGAGAAGA
